# *Period 2*: A Regulator of Multiple Tissue-Specific Circadian Functions

**DOI:** 10.3389/fnmol.2021.718387

**Published:** 2021-09-03

**Authors:** Gennaro Ruggiero, Zohar Ben-Moshe Livne, Yair Wexler, Nathalie Geyer, Daniela Vallone, Yoav Gothilf, Nicholas S. Foulkes

**Affiliations:** ^1^Institute of Biological and Chemical Systems, Karlsruhe Institute of Technology, Karlsruhe, Germany; ^2^Department of Physiology, Anatomy and Genetics, University of Oxford, Oxford, United Kingdom; ^3^School of Neurobiology, Biochemistry and Biophysics, Faculty of Life Sciences, Tel Aviv University, Tel Aviv, Israel; ^4^Sagol School of Neuroscience, Tel Aviv University, Tel Aviv, Israel

**Keywords:** circadian clock, zebrafish, period, cell cycle, behavior, metabolism

## Abstract

The zebrafish represents a powerful model for exploring how light regulates the circadian clock due to the direct light sensitivity of its peripheral clocks, a property that is retained even in organ cultures as well as zebrafish-derived cell lines. Light-inducible expression of the *per2* clock gene has been predicted to play a vital function in relaying light information to the core circadian clock mechanism in many organisms, including zebrafish. To directly test the contribution of *per2* to circadian clock function in zebrafish, we have generated a loss-of-function *per2* gene mutation. Our results reveal a tissue-specific role for the *per2* gene in maintaining rhythmic expression of circadian clock genes, as well as clock-controlled genes, and an impact on the rhythmic behavior of intact zebrafish larvae. Furthermore, we demonstrate that disruption of the *per2* gene impacts on the circadian regulation of the cell cycle *in vivo*. Based on these results, we hypothesize that in addition to serving as a central element of the light input pathway to the circadian clock, *per2* acts as circadian regulator of tissue-specific physiological functions in zebrafish.

## Introduction

The circadian clock is an endogenous and self-sustaining timing mechanism present in most organisms, which evolved to anticipate daily environmental changes and thereby to coordinate physiological and behavioral adaptations (Pittendrigh, 1993). Consistent with its central coordinating role within physiology, disruption of the circadian timing system is associated with many pathological conditions (Toh et al., 2001; Turek et al., 2005; Savvidis and Koutsilieris, 2012). A vital feature of the internal clock is that external environmental signals (*zeitgebers*, primarily light, but also food and temperature changes) can regularly adjust the phase of the circadian system to ensure synchronization with the environmental day-night cycle.

In vertebrates, at the core of the molecular mechanism of the circadian clock is a series of interlocking transcription-translation feedback loops. The positive limb of these regulatory loops is constituted by the transcription factors CLOCK and BMAL, which heterodimerize, bind to E-box enhancer promoter elements and thereby activate the transcription of downstream clock target genes. These include genes which constitute the negative limb of the clock mechanism, the period (*Per*) and cryptochrome (*Cry*) genes. Following translation, the PER and CRY proteins heterodimerize, translocate back to the nucleus, and inhibit transcriptional activation directed by CLOCK/BMAL (Partch et al., 2014). CLOCK and BMAL also regulate the expression of other, clock-controlled genes (CCGs) including the transcription factors *Rev-erbα* and *Rorα* which form a stabilizing regulatory loop within the core clock mechanism.

Over the course of vertebrate evolution, the regulatory mechanisms, as well as the anatomical organization which underlies the circadian timing system, have undergone several changes (Menaker et al., 1997). At the anatomical level, in mammals the circadian timing system is characterized by a “master” clock located in the suprachiasmatic nucleus (SCN) of the hypothalamus with multiple independent “peripheral” clocks distributed in most tissues, organs and cells. This SCN clock receives light input indirectly from the retina and is thereby synchronized with the external solar day. It subsequently relays this timing information to the peripheral clocks via a variety of endocrine and systemic cues (Schibler and Sassone-Corsi, 2002; Schibler et al., 2015). In contrast, in non-mammalian vertebrates, a directly light-entrainable circadian oscillator is distributed in multiple tissues including the pineal gland, retina, and various brain nuclei, predicting the widespread expression of photoreceptors and elements of the clock light input pathway (Fukada and Okano, 2002). An extreme independence of central clock regulation can be seen in teleost fish where all peripheral clocks can be directly light-entrained (Whitmore et al., 1998; Sassone-Corsi et al., 2000; Foulkes, 2016). At the genomic level, several clock genes have undergone duplications and probably species-specific and tissue-specific sub-functionalization. The *per* gene has three homologs in mammals and four homologs in fish, *per1a*, *per1b*, *per2*, and *per3*.

The *per2* gene has been predicted to play an important role in the photic entrainment mechanism of the circadian clock in the vertebrate circadian timing mechanism. Indeed, levels of *per1* and *per2* mRNA expression are transiently induced in response to light exposure, in the mouse SCN (Albrecht et al., 1997; Bae et al., 2001). Furthermore, in zebrafish the expression of the *per2* gene is induced robustly following direct exposure of intact larvae, isolated tissues, cells and even cell lines to light via the effect of a D-box enhancer promoter element located in the *per2* gene promoter region (Vatine et al., 2009; Mracek et al., 2012). In addition to light-entrainment, PER2 is linked with the clock mechanism itself, as the S752G PER2 mutation in humans leads to hypo-phosphorylation, PER2 stabilization, and a familial advanced sleep-phase syndrome (FASPS) phenotype (Toh et al., 2001). Via its ability to downregulate transactivation driven by the CLOCK/BMAL complex within the core clock mechanism, the PER2 protein appears to contribute to the circadian regulation of a wide range of cellular functions (Albrecht et al., 2007), including metabolism and cell cycle (Fu et al., 2002; Grimaldi et al., 2010; Gu et al., 2012).

Several studies have also pinpointed a direct role for the PER2 protein, independent of its function within the core circadian clock. Thus, in zebrafish, the PER2 protein has been implicated in the direct transcriptional regulation of the *bmal1* gene via the retinoic acid—related orphan receptor response element (RORE) binding sites in zebrafish (Wang et al., 2015). Furthermore, we have shown that light-induced expression of *per2* during early embryonic development is a prerequisite for the development of a functional circadian clock system (Ziv and Gothilf, 2006). In addition, in mouse, it has been implicated as a tumor suppressor gene (Fu et al., 2002). Therefore, current evidence points to this clock protein playing a diverse role in the dynamic control of physiological systems, including the cell cycle.

In this report, we have explored the function of the *per2* clock gene in zebrafish by generating a new *per2* knockout (KO) zebrafish line. Specifically, using TALEN technology, we introduced a truncation mutation into the zebrafish *per2* locus, and then characterized the resulting phenotype of the *per2* KO zebrafish line. We show that loss of *per2* gene function results in an abnormal pattern of rhythmic locomotor activity in *per2* KO larvae under different lighting conditions and conclude that *per2* plays an essential role in the regulation of circadian phase and amplitude of behavioral rhythms and their entrainment by light. Moreover, we demonstrate a tissue-specific function for the *per2* gene in the maintenance of rhythmic expression of core circadian clock genes and CCGs. Finally, we reveal that disruption of *per2* gene function impacts on circadian regulation of the cell cycle *in vivo*. Therefore, these results point to a pleiotropic function for the *per2* gene in circadian regulation of tissue specific function.

## Materials and Methods

### Animals

Wild type (WT) and corresponding *per2* KO sibling AB strain zebrafish lines were raised at 28°C under a 14 h:10 h light/dark cycle from the hatching stage. Lights were turned on at 8:00 and turned off at 22:00 and the fish were fed twice daily. To generate embryos, male and female zebrafish were paired in the evening, and spawning occurred the next day within 1 h after lights on. For locomotor activity analysis, embryos were transferred into 48-well plates (one larva per well) during the 4th or 5th day of development and placed into the DanioVision observation chamber (Noldus Information Technology). All zebrafish procedures were approved by the Tel-Aviv University Animal Care Committee (04-18-051) and conducted in accordance with the National Council for Animal Experimentation, Ministry of Health, Israel. At the Karlsruhe Institute of Technology, all husbandry and experimental procedures were performed in accordance with European Legislation for the Protection of Animals used for Scientific Purposes (Directive 2010/63/EU), the German Animal Protection Law [May 18th, 2006 (BGBl. I S. 1206, 1313), last changed March 29th, 2017 (BGBl. I S. 626)]. Research was also approved by the Local Government of Baden-Württemberg, Karlsruhe, Germany (35-9185.81/G-131/16 and 35-9185.82/A-9/18). General license for fish maintenance and breeding: Az.: 35-9185.64.

### Generation of *per2* KO Fish

**FIGURE 1:**
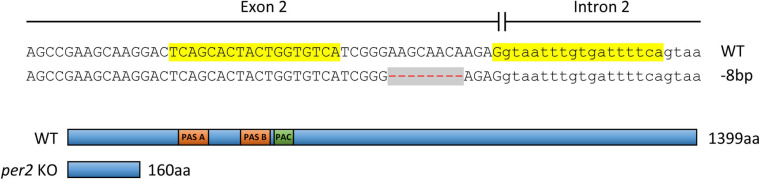
**Above:** Establishment of a TALEN-mediated *per2* KO zebrafish line. A pair of TALENs was used to target the 2nd exon of the zebrafish *per2* gene (TALEN left and right target sites are highlighted in yellow). A deletion of 8 bp (red dashes against a gray background) resulted in a frameshift mutation. **Below:** The consequent introduction of a premature stop codon and a predicted truncated PER2 protein of 160 aa compared with the 1399 aa WT protein. PER-ARNT-SIM domains (PAS, orange) and C-terminal of PAS domain (PAC, green) in the WT protein are indicated.

Genome editing with the transcription activator-like effector nucleases (TALEN) system was used to generate *per2* KO fish, registered in the Zebrafish Model Organism Database (ZFIN) as *per2*^tlv02^. Specific TALENs designed to target the 2nd exon of *per2* (TALE F target sequence 5′-tcagcactactggtgtca-3′, TALE R target sequence 5′-tgaaaatcacaaattacc-3′) were obtained from Addgene (TAL3138 and TAL3139, Addgene plasmids #41312 and #41313). The TALE nuclease expression vectors were linearized with PmeI and transcribed using mMESSAGE mMACHINE T7 kit (Ambion) followed by the Poly-A tailing kit (Ambion) according to the manufacturer’s protocol. Approximately 2 nl of the TALENs mRNA at concentration of 100 ng/μl each were microinjected into one-cell stage embryos (F0). The injected embryos were raised, and their progeny (F1) were fin-clipped and screened by PCR (using the primers: per2-E2-F 5′-gccagtttcgcagaaggcactg-3′, per2-I2-R 5′-agccatcaggtctcaactgtttgtca-3′) followed by T7E1 assay for identifying mutations in *per2* coding sequence. A male and female F1 fish carrying the same 8 bp deletion mutation in *per2* exon 2 (Figure 1) were identified by sequencing and crossed to produce homozygous KO fish (F2) and their WT siblings. The progeny of the F2 mutated homozygotes and of their WT siblings were used for behavioral analysis, raising a possibility of observing maternal effects of the mutation. However, the lack of any conclusive evidence for maternal inheritance of circadian clock function in zebrafish makes such a maternal effect unlikely (Whitmore et al., 1998).

### Locomotor Activity Monitoring of Zebrafish Larvae and Statistical Analysis

**FIGURE 2:**
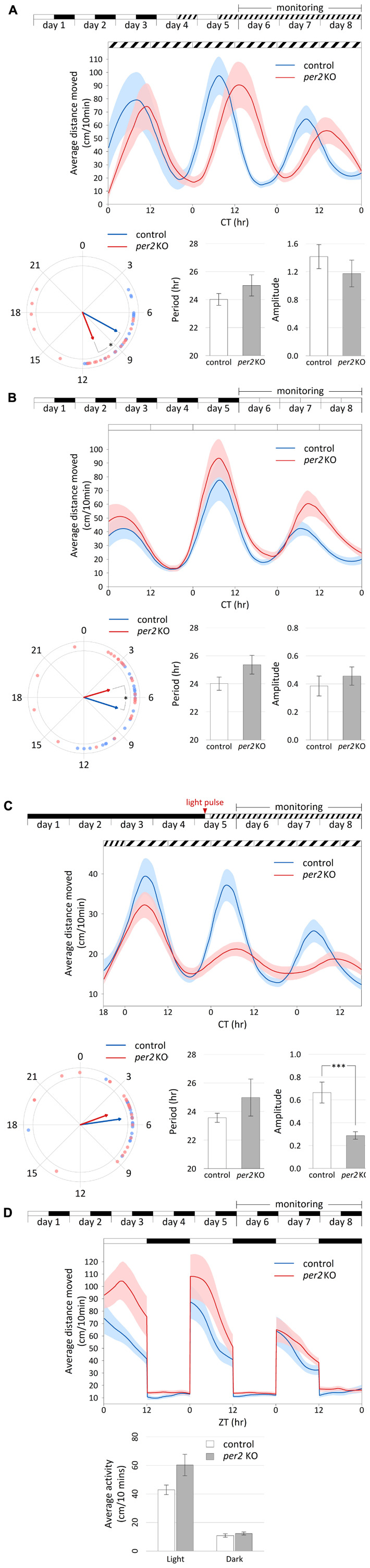
*Per2* KO affects the phase of circadian rhythms of locomotor activity and their entrainment by light. Analysis of locomotor activity of 6–8 dpf *per2* KO and control larvae under various lighting conditions. **(A–D)** Top, experimental design of the photic treatment prior to and throughout activity monitoring. White boxes represent light, black boxes represent dark, and diagonally lined boxes represent dim light. Middle, the average distance moved (cm/10 min) is plotted on the *y*-axis and circadian time (CT) for panels **(A–C)** or *zeitgeber* time (ZT) for panel **(D)** is plotted on the *x*-axis; error bars indicate SE. **(A–C)** Bottom from left to right, comparison of average phase, period (±SE) and amplitude (±SE) between genotypes. In the circular plot of circadian phase, arrow direction represents the average phase for each genotype and the length represents the variance (longer arrow stands for low variance and vice versa). **(D)** Bottom, comparison of average activity (±SE) between genotypes throughout the light and dark segments. **(A)** Circadian rhythms of locomotor activity under DimDim, after entrainment by 3 LD cycles and 2 light-dim light (LDim) cycles; *per2* KO larvae (*n* = 21) display a phase delay of 2.7 h compared to control larvae (*n* = 20; *p* < 0.05 (denoted by *), Watson–Williams test). **(B)** Circadian rhythms of locomotor activity under LL, after entrainment by 5 LD cycles; *per2* KO larvae (*n* = 24) display a phase advance of 2.3 h compared to control larvae (*n* = 22; *p* < 0.05 (denoted by *), Watson–Williams test). **(C)** Circadian rhythms of locomotor activity under DimDim, after exposure to a 3-h light pulse (indicated by red arrowhead); *per2* KO larvae (*n* = 23) display a decreased amplitude of activity rhythmicity compared to control larvae (*n* = 23; *p* < 0.001 (denoted by ***), *t*-test). **(D)** Locomotor activity under LD cycles is not affected by *per2* KO; no significant difference in average activity was observed between genotypes during both the light and the dark segments (*n* = 24 *per2* KO; *n* = 23 controls).

Homozygous *per2* KO embryos and control embryos (progeny of WT siblings) were raised in a light- and temperature-controlled incubator under 12-h:12-h LD cycles or constant darkness at 28°C. On the 4th or 5th day of development, larvae were placed in 48-well plates in the observation chamber of the DanioVision tracking system (Noldus Information Technology) for acclimation under controlled temperature (28°C) and lighting conditions (LED; intensity of “light” and “dim light” were 1.8 W/m^2^ and 0.013 W/m^2^, respectively) according to the desired protocol. Starting from the 6th day of development, movement was tracked and analyzed by the EthoVision XT 11 software (Noldus Information Technology). Locomotor activity was measured across three daily cycles by the total distance moved (cm) by each larva per 10 min time-bins. All experiments were repeated independently two to four times, the results shown in Figure 2 are of one representative experiment.

For the analysis of circadian activity under constant conditions (Figures 2A–C), normalization of the data was obtained by dividing each activity value by the mean activity value. Short-term trends were removed by a LOESS-smoothed 75th-percentile function (Benjamini et al., 2011) with half window widths of 3.33 h (20 sliding points) for the moving percentiles and 8.33 h (50 sliding points) for the LOESS curve. Peaks (local maxima) and troughs (local minima) in the normalized smoothed curves were used to compute the period, phase and amplitude of the circadian locomotor activity rhythms. Period was estimated as the weighted mean time difference (in hr) between each pair of consecutive peaks. Phase was estimated as the weighted mean direction [mean of circular quantities; (Jammalamadaka and SenGupta, 2001)] of peak time relative to the estimated period. Since higher amplitudes are less sensitive to noise, weight was assigned to each peak in proportion to its amplitude for estimating period and phase. The reported amplitude was defined as half the difference in normalized activity between the peak of the 2nd day of tracking and the preceding trough. Values are reported as mean ± standard error (SE). Statistical differences in period and amplitude between groups were determined by *t*-test, and statistical differences in phase were determined by Watson–Williams test for the homogeneity of means.

In an experiment with LD cycles (Figure 2D), smoothing was performed separately on each cycle with half window widths of 2.5 h (15 sliding points) for the moving percentiles and 4.17 h (25 sliding points) for the LOESS curve. In order to assess the difference in activity between groups under the light and dark conditions, the average distance moved (cm/10 min) was calculated for each larva separately for the light and for the dark segments. The activity values (log-transformed) of the two genotypes under the light and dark segments were compared by mixed model ANOVA.

Data plotted in Figures 2A–D (middle chart) is the average across larvae ± SE of the non-normalized LOESS-smoothed percentile functions, with each group consisting of 20–24 larvae.

### Gene Expression Analysis

Total RNA of zebrafish tissues was extracted using TRIzol reagent (Invitrogen) according to the manufacturer’s instructions. The concentrations of RNA samples were assessed with a NanoDrop ND-1000 spectrometer (PeqLab). The quality of the RNA was determined after electrophoresis on an agarose gel to visualize the integrity of the ribosomal 28S, 18S, and 5S RNA bands. The first strand cDNA synthesis of total RNA was performed according to the manufacturer’s protocol (Promega). Quantitative PCR was performed using the Step One Plus Real-Time PCR System (Applied Biosystems) and SYBR Green (Promega) master mix according to the manufacturers’ recommendations. Primer sequences are shown in Supplementary Table 1. The relative expression levels for each gene were calculated by the 2^–ΔΔCT^ method and normalized using the relative expression of β-actin.

### Western Blotting

Protein extracts were prepared by homogenizing sample tissues in 1X Laemmli (6% SDS, 20% glycerol, 125 mM Tris pH 6.8, 0.01% bromophenol blue, 100 mM DTT) containing a 1X cocktail of protease inhibitors (Sigma-Aldrich) buffer. The samples were electrophoresed on an SDS polyacrylamide gel and transferred to an Immun-Blot [polyvinylidene difluoride (PVDF)] membrane (Millipore) by electroblotting. Antibody incubation and washing was performed following the manufacturers’ recommendations and visualization was performed by using the ECL detection system (Bio-Rad). Images were acquired and analyzed by using the Image Lab Software (Bio-Rad).

### Statistical Analysis of Gene Expression

Significance in the difference in gene expression dynamics between mutants and controls was assessed via a two-way ANOVA, fitted independently for each gene in each tissue. The ANOVA model consisted of three fixed effects: genotype (*per2* KO or WT siblings), time, and their interaction genotype × time (see detailed results in Table 1). A significant interaction indicates an alteration in the gene’s expression dynamics in the mutants. A significant genotype effect indicates a total increase or reduction in expression level in the mutants across all time points. A significant time effect indicates non-constant expression over time as would be expected in the rhythmic genes chosen in this work. *Post hoc* analysis was performed in instances of significant interaction (*p* < 0.05), comparing the two genotypes at each time point individually. *P*-values were corrected using Sidak’s method. Analysis was carried out using GraphPad Prism 7.0. All estimates are expressed as means ± SD of biological or technical replicates.

**TABLE 1:** Under LD conditions, the *per2* knockout alters circadian rhythms of mRNA expression in (i) CCGs in the liver, heart, fins, muscles, gut and eyes (Figure 4), (ii) clock-controlled genes in the heart (Figure 5), (iii) regulators of key physiological hepatic processes in the liver (Figure 6), (iv) genes encoding enzymes involved in biosynthesis of non-essential amino acids in the liver (Figure 7), (v) regulators of skeletal muscle myogenesis and regeneration in the muscles (Figure 8), and in (vi) cell-cycle regulators in the fin (Figure 9).

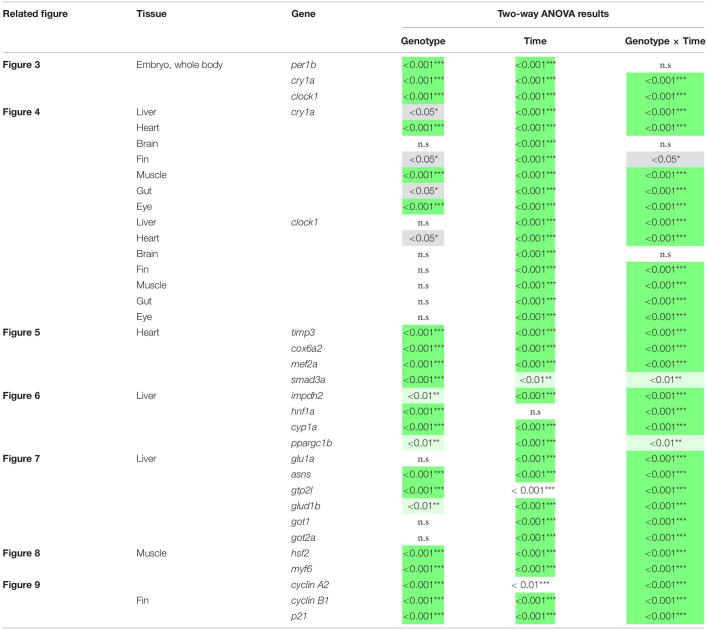

**FIGURE 3:**
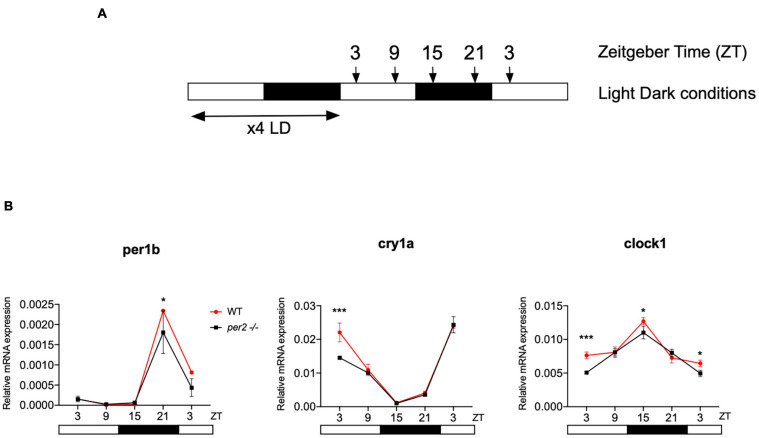
Circadian clock gene expression analysis in *per2* KO and WT larvae. **(A)** Schematic representation of the experimental design. The horizontal bars represent the lighting conditions before and during sampling; white and black boxes indicate light and dark periods, respectively; the arrows show the sampling times. WT and *per2* mutant larvae were kept 4 dpf in LD cycle (12 h light-12 h dark) conditions. On the 5th dpf, total RNA was extracted from pools of larvae collected at 6-h intervals during a sampling window of 24 h. **(B)** qRT-PCR analysis of mRNA expression levels of the circadian clock genes (*clock1*, *cry1a*, and *per1b*) in WT and *per2* mutant larvae. Mean mRNA relative expression (*n* = 2–3) ± SD is plotted on the *y*-axis, while *zeitgeber* time (ZT) is plotted on the *x*-axis. ZT0 corresponds to lights-on, ZT12 to lights-off. A significant, small decrease in expression level was observed in mutants in all three genes (***p < 0.001, ANOVA genotype effect, Table 1). Asterisks represent levels of significance in comparing the two genotypes at each time point individually, corrected by Sidak’s method (***p < 0.001, *p < 0.05).

qRT-PCR was used to measure mRNA expression levels across five time points at 6 hourly intervals. Significance in the difference in gene expression dynamics between mutants and controls was assessed via a two-way ANOVA, fitted independently for each gene in each tissue. The ANOVA model consisted of three fixed effects: genotype (*per2* KO or WT siblings), time, and their interaction genotype × time. A significant interaction indicates an alteration in the gene’s expression dynamics in the mutants. A significant genotype effect indicates difference in the gene’s average expression between the mutants and the controls across all time points. A significant time effect was exhibited in all tissues in almost all genes, indicating unsurprisingly that the expression over time is non-constant. The exception is *hnf1a* in the liver (Figure 6), whose rhythmic expression was maintained in the mutants, but with a 12 h phase-shift. No effect of *per2* knockout on the circadian rhythm was observed in whole-body mRNA expression of the CCGs *per1*, *cry1a* and *clock1* in embryos (Figure 3), as well as expression of *cry1a* and *clock1* in adult brains. Darker green shading donotes calculated p values of <0.001, light green shading denotes *p* < 0.01 and grey shading denotes *p* < 0.05 (***p < 0.001, **p < 0.01, *p < 0.05).

## Results

### Altered Rhythmic Locomotor Activity in *per2* Mutant Larvae

In order to directly address the function of *per2* in zebrafish, we established a TALEN-based *per2* KO fish line. An 8 bp deletion was generated in the 2nd exon of *per2*, resulting in a frameshift which is predicted to encode a truncated protein of 160 amino acids (aa) instead of the 1399 aa WT PER2 protein (Figure 1). Without the crucial PAS protein-protein interaction domains, this small, truncated protein is predicted to lack the normal function that involves direct interaction with other transcriptional regulatory factors. The presence of this mutation was subsequently validated by PCR and DNA sequencing in each subsequent generation of the *per2* KO line.

In order to assess the impact of this TALEN-generated mutation on clock-regulated behavioral rhythms and their entrainment by light, we initially analyzed the rhythmic locomotor activity of *per2* KO and control larvae under different photic regimes. Following entrainment by LD cycles (Cahill et al., 1998) or by exposure to a single pulse of light (Ben-Moshe et al., 2014), WT larval zebrafish display daily rhythms of locomotor activity under constant conditions with higher activity levels during the subjective daytime, a pattern that is highly reproducible amongst independently raised families.

Locomotor activity was measured in LD-entrained *per2* KO and WT control larvae that were placed under constant dim light (DimDim; Figure 2A) or under constant light (LL; Figure 2B). Under both conditions, rhythms of locomotor activity were maintained with no significant difference in period or amplitude (DimDim, periods of 24.0 ± 0.4 and 25.0 ± 0.8 and amplitudes of 1.42 ± 0.17 and 1.17 ± 0.19 for control and *per2* KO larvae, respectively; LL, periods of 24.0 ± 0.5 h and 25.4 ± 0.7 h and amplitudes of 0.38 ± 0.07 and 0.46 ± 0.07 for control and *per2* KO larvae, respectively). However, substantial phase differences between the genotypes were observed under both conditions. Interestingly, the two lighting conditions induced an opposite effect. While under DimDim, *per2* KO larvae exhibited a phase delay of 2.7 h compared to the controls, under LL, *per2* KO larvae exhibited a phase advance of 2.3 h (DimDim, phases of 7.9 ± 0.5 and 10.6 ± 0.8 for control and *per2* KO larvae, respectively; *p* < 0.05, Watson–Williams test; LL, phases of 7.2 ± 0.6 and 4.9 ± 0.8 for control and *per2* KO larvae, respectively; *p* < 0.05, Watson–Williams test). Thus, loss of *per2* function induces a differential effect on the phase of locomotor activity rhythms which depends on the lighting conditions. The results under DimDim (Figure 2A) agree with a previous report with a different *per2* mutant, in which *per2* mutant larvae presented an approximately 2-h phase delay and a ∼1.1-h lengthened period under constant darkness (Wang et al., 2015).

When larvae raised in constant darkness were exposed to a single 3-h light pulse on the 5th day post-fertilization (dpf) and then monitored under DimDim (Figure 2C), a procedure which is sufficient to trigger and set the phase of high amplitude rhythms of locomotor activity (Ben-Moshe et al., 2014), *per2* KO larvae displayed a significantly lower amplitude of activity (0.66 ± 0.09 and 0.29 ± 0.03 for control and *per2* KO larvae, respectively, *p* < 0.001, *t*-test). However, the period and phase did not change significantly (periods of 23.6 ± 0.3 h and 25.0 ± 1.3 h and phases of 5.5 ± 0.4 and 4.7 ± 0.8 for control and *per2* KO larvae, respectively). These observations reflect the predicted role of PER2 in light-entrainment and are consistent with previous findings obtained by morpholino-mediated *per2* knock-down using a similar experimental setup (Ben-Moshe et al., 2014), where circadian locomotor activity rhythms were similarly disrupted by this manipulation.

Under LD cycles (Figure 2D), the locomotor activity patterns of *per2* KO larvae were unaltered compared to control larvae. Both groups exhibited significantly higher activity during the light phase compared to the dark phase (*p* < 0.0001, ANOVA) due to a masking effect, in which activity is mainly determined by the lighting conditions and not by the clock. No significant differences were observed between the activity of the two groups during both the light and the dark segments, an observation that does not correspond to a previously reported experiment with another *per2* mutant, where *per2* mutant larvae displayed reduced overall locomotor activity under LD conditions compared to control larvae (Wang et al., 2015). This dissimilar outcome from two *per2* mutant lines may reflect different experimental conditions or genetic backgrounds. Overall, our analysis supports an essential role for PER2 in the regulation of circadian phase and amplitude of behavioral rhythms and their entrainment by light.

### Expression of Circadian Clock Genes in *per2* Mutant Zebrafish Larvae

In order to explore the consequences of loss of *per2* function at the gene expression level, we examined the pattern of rhythmic core clock gene expression in the *per2* mutants under normal LD (12 h light-12 h dark) cycle conditions. We initially compared the dynamic mRNA expression pattern of a subset of circadian clock genes (*clock1*, *cry1a*, and *per1b*) in whole body RNA extracts of WT sibling and *per2* mutant zebrafish larvae raised under LD cycle conditions (Figure 3). While our results revealed a small reduction in expression levels in the mutants in the case of all 3 clock genes, the overall expression pattern for these clock genes was comparable in the WT and mutant larvae.

### Tissue-Specific Regulatory Roles of *per2* in Adult Zebrafish Peripheral Tissues

**FIGURE 4:**
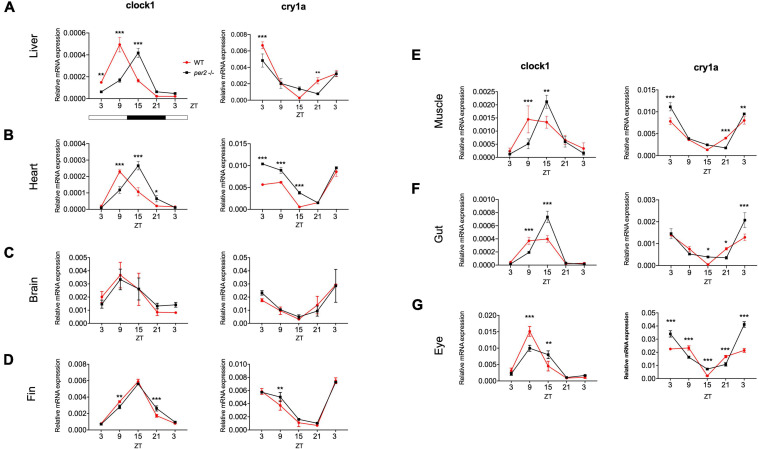
*Per2* knockout alters rhythmic mRNA expression of the *cry1a* and *clock1* clock genes in adult zebrafish tissues. qRT-PCR analysis of expression levels of the circadian clock genes *clock1* and *cry1a* in the **(A)** liver, **(B)** heart, **(C)** brain, **(D)** fin, **(E)** muscle, **(F)** gut and **(G)** eyes of WT and *per2* mutant adult fish. Sets (*n* = 3) of 4 WT and *per2* KO fishes (each set containing two males and two females) were maintained under LD cycle (14 h light-10 h dark) conditions, tissues were dissected and pooled for RNA extraction at 6-h intervals during a sampling window of 24 h. Mean mRNA relative expression ± SD is plotted on the *y*-axis; *zeitgeber* time (ZT) is plotted on the *x*-axis. *Zeitgeber* times are indicated for each sample. ZT0 corresponds to lights-on, ZT14 to lights-off. A change in rhythm was observed for both genes in liver, heart, fin, muscle, gut, and eyes, as determined by a significant ANOVA Genotype × Time interaction effect (*p* < 0.001, see Table 1). For both genes, no effect was observed in the brain. Asterisks represent levels of significance in comparing the two genotypes at each time point individually, corrected by Sidak’s method (***p < 0.001, **p < 0.01, *p < 0.05).

Since the whole-body expression pattern of clock genes did not differ greatly between *per2* mutants and WT fish, we next tested for tissue-specific differences in the expression of clock genes. We examined the rhythmic expression of the *cry1a* and *clock1* clock genes in liver, heart, brain, fin, muscle, gut, and eyes of WT and *per2* mutant zebrafish adults maintained under LD cycle conditions (Figure 4). Interestingly, a change in the rhythmic profile of *clock1* and *cry1a* expression was detected in the heart, liver, gut and muscle of the *per2* mutants relative to the WT fish.

Given the observation of tissue-specific changes in the rhythmic expression of certain clock genes in our zebrafish mutant, we next performed a gene expression analysis of CCGs in *per2* KO zebrafish heart, liver, and muscle under normal LD cycle conditions, as a first step toward evaluating the possible impact of the *per2* mutation on zebrafish cardiac, hepatic and skeletal muscle physiology.

**FIGURE 5:**
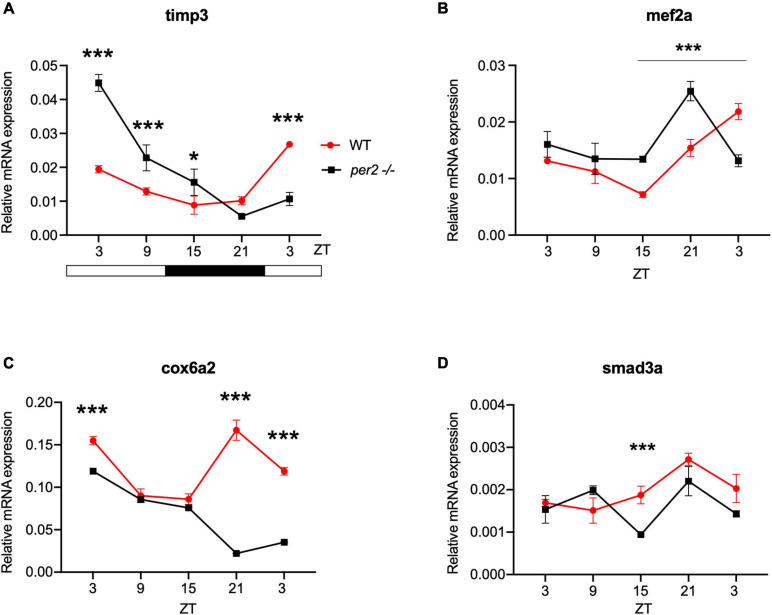
*Per2* knockout alters the rhythmic mRNA expression of clock-controlled genes in the adult zebrafish heart. qRT-PCR analysis of expression levels of four putative CCGs **(A)** *timp3*, **(B)** *mef2a*, **(C)** *cox6a2*, and **(D)** *smad3a* in WT and *per2* KO heart tissues. Sets (*n* = 3) of 4 WT and *per2* KO fish (each set containing two males and two females) were maintained under LD cycle (14 h light-10 h dark) conditions, hearts were dissected and pooled for RNA extraction at 6-h intervals during a sampling window of 24 h. Mean mRNA relative expression ± SD is plotted on the *y*-axis; *zeitgeber* time (ZT) is plotted on the *x*-axis. *Zeitgeber* times are indicated for each sample. ZT0 corresponds to lights-on, ZT14 to lights-off. A change in rhythm was observed for *timp3*, *cox6a2, mef2a*, and *smad3a*, as determined by a significant ANOVA Genotype × Time interaction effect for all genes (*p* < 0.01, see Table 1). Asterisks represent levels of significance in comparing the two genotypes at each time point individually, corrected by Sidak’s method (***p < 0.001, *p < 0.05).

**FIGURE 6:**
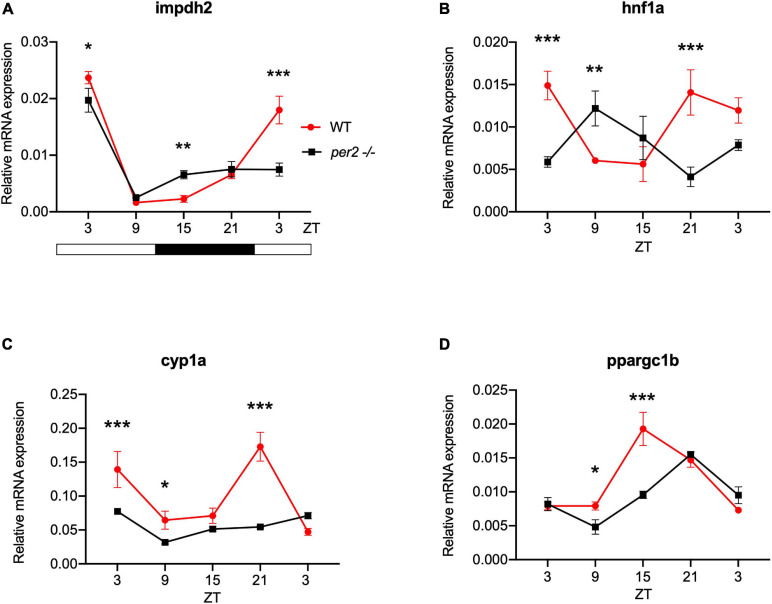
*Per2* knockout alters the rhythmic mRNA expression of regulators of key physiological hepatic processes in the adult zebrafish liver. qRT-PCR analysis of expression levels of four CCGs **(A)** *impdh2*, **(B)** *hnf1a*, **(C)** *cyp1a*, and **(D)** *ppargc1b* in WT and *per2* KO liver. Sets of 4 WT and *per2* KO fishes (each set containing two males and two females) were maintained under LD cycle (14 h light-10 h dark) conditions, livers were dissected and pooled for RNA extraction at 6-h intervals during a sampling window of 24 h. Mean mRNA relative expression (*n* = 2–3) ± SD is plotted on the *y*-axis; *zeitgeber* time (ZT) is plotted on the *x*-axis. *Zeitgeber* times are indicated for each sample. ZT0 corresponds to lights-on, ZT14 to lights-off. A change in rhythm was observed for *impdh2*, *hnf1a, cyp1a* and *ppargc1b*, as determined by a significant ANOVA Genotype × Time interaction effect for all genes (*p* < 0.01, see Table 1). Furthermore, *hnf1a* exhibited a 12 h phase delay, and *ppargc1b* exhibited a 6 h phase delay. Asterisks represent levels of significance in comparing the two genotypes at each time point individually, corrected by Sidak’s method (***p < 0.001, **p < 0.01, *p < 0.05).

**FIGURE 7:**
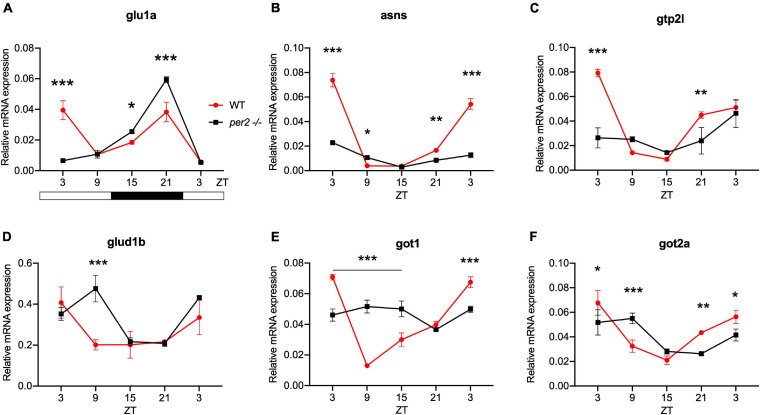
*Per2* knockout alters the rhythmic mRNA expression of CCGs encoding key enzymes in non-essential amino acid synthesis in the adult zebrafish liver. qRT-PCR analysis of expression levels of six CCGs **(A)** *glu1a*, **(B)** *asns*, **(C)** *gpt2l* **(D)** *glud1b* **(E)** *got1*, and **(F)** *got2a* in WT and *per2* KO liver tissues as described in the legend for Figure 6. A change in rhythm was observed for all genes, as determined by a significant ANOVA Genotype × Time interaction effect for all genes (*p* < 0.001, see Table 1). Asterisks represent levels of significance in comparing the two genotypes at each time point individually, corrected by Sidak’s method (***p < 0.001, **p < 0.01, *p < 0.05).

For the heart, we examined the expression of the following CCGs: *timp3*, encoding a tissue inhibitor of matrix metalloproteinases that was identified as being circadian clock regulated in the mouse heart (Durgan and Young, 2010), *mef2a* a clock controlled transcription factor involved in heart development and myofibril assembly (Wang et al., 2005, 2007), *cox6a2* a nuclear-encoded cytochrome oxidase subunit involved in mitochondrial electron transport, that shows circadian modulation in the mouse heart (Martino et al., 2004) and *smad3a*, a TGF-β signaling gene exhibiting a circadian expression pattern throughout the brain of zebrafish larvae (Sato et al., 2012; Sloin et al., 2018; Finger et al., 2021). The circadian expression of each of these CCGs in *per2* KO heart tissue was affected, with a significant alteration of the circadian expression pattern, thus suggesting a potential involvement of *per2* in the circadian regulation of heart physiology in zebrafish (Figure 5). We then analyzed the expression of the *impdh2* [IMP dehydrogenase, a rate limiting enzyme in *de novo* purine synthesis (Li et al., 2013)], *cyp1a* [cytochrome P4501A, involved in detoxification (Carmona-Antoñanzas et al., 2017)], *ppargc1b* [Peroxisome proliferator-activated receptor gamma coactivator 1-beta, a transcriptional coactivator involved in multiple aspects of cellular energy metabolism (Lin et al., 2003)] and *hnf1a* [hepatocyte nuclear factor 4, alpha, involved in regulating liver-specific gene expression (Courtois et al., 1987)] genes which all encode regulators of key physiological hepatic processes and have been reported to show circadian rhythms of expression in zebrafish larvae (Li et al., 2013). The *cyp1a*, *ppargc1b*, and *hnf1a* genes all exhibited a significantly altered circadian expression pattern in the *per2* KO liver (Figure 6). Specifically, *cyp1a* showed a general reduction in expression levels and disrupted rhythmic expression. Furthermore, *ppargc1b* and *hnf1a* exhibited a 6 and 12 h phase delay of the rhythmic pattern, respectively. Interestingly, however, the phase of rhythmic expression of *impdh2* resembled that observed in WT liver controls, suggesting that the precise pattern of disrupted rhythmic gene expression upon loss of *per2* function differs between CCGs. Given the central role played by the circadian clock in regulating key metabolic pathways including amino acid biosynthesis (Krishnaiah et al., 2017), we next chose to test if the circadian expression of a set of CCGs encoding key or rate-limiting enzymes involved in the biosynthetic pathways for non-essential amino acids (Li et al., 2013), was affected in the *per2* KO liver. We specifically tested expression of *got1* and *got2a* (glutamic-oxaloacetic transaminase 1, and 2a) which are linked with the aspartate biosynthesis pathway), asparagine synthetase (*asns*) mediating asparagine production, glutamate dehydrogenase 1b (*glud1b*), mediating glutamate synthesis, glutamine synthetase 1a (*glu1a*), catalyzing glutamine formation and glutamic pyruvate transaminase 2-like (*gpt2l*), involved in alanine biosynthesis. Significant changes in the pattern of rhythmic expression of all six CCGs were observed in *per2* KO liver (Figure 7). Specifically, *gtp2l, asns*, and *got1* all displayed a reduced amplitude of rhythmic expression. Furthermore, *glud1b, asns*, *got1*, and *got2a* exhibited a phase shift of approximately 6 h, as previously observed for clock gene expression.

**FIGURE 8:**
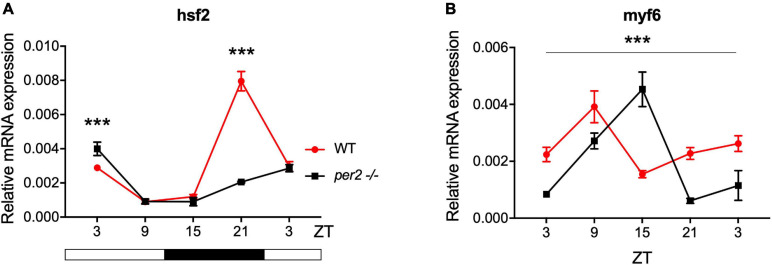
*Per2* knockout alters the rhythmic mRNA expression of CCGs in adult zebrafish skeletal muscle. qRT-PCR analysis of expression levels of the CCGs **(A)** *hsf2* and **(B)** *myf6* in WT and *per2* KO skeletal muscle. Sets of 4 WT and *per2* KO fish (each set containing two males and two females) were maintained under LD cycle (14 h light-10 h dark) conditions, muscle tissue was dissected and pooled for RNA extraction at 6-h intervals during a sampling window of 24 h. Mean mRNA relative expression (*n* = 2–3) ± SD is plotted on the *y*-axis; *zeitgeber* time (ZT) is plotted on the *x*-axis. *Zeitgeber* times are indicated for each sample. ZT0 corresponds to lights-on, ZT14 to lights-off. A change in rhythm was observed for both CCGs, as determined by a significant ANOVA Genotype × Time interaction effect for all genes (*p* < 0.001, see Table 1). *myf6* exhibited a 6 h phase delay. Asterisks represent levels of significance in comparing the two genotypes at each time point individually, corrected by Sidak’s method (***p < 0.001).

Expression of CCGs in the *per2* KO skeletal muscle was also tested. In particular, based on a previous study that identified putative CCGs in skeletal muscle of the zebrafish (Amaral and Johnston, 2012), we tested the expression of *myf6* and *hsf2* (Figure 8), that play an important role during skeletal muscle myogenesis and regeneration (McArdle et al., 2006; Hinits et al., 2007; Wang et al., 2008; Chong et al., 2009; Amaral and Johnston, 2012). We revealed that both *hsf2* and *myf6* exhibited a significant alteration in their rhythmic expression pattern in *per2* KO skeletal muscle. Rhythmic *Myf6* expression showed a phase delay of 6 h that matches the phase shift observed in the expression pattern of the clock genes *cry1a* and *clock1* while *hsf2* exhibited a robust reduction of rhythm amplitude. Thus, taken together, our findings (Figures 4–8) implicate the *per2* gene in playing a role in circadian clock regulation in a tissue- and gene-specific manner.

### Abnormal Cell Cycle Control in *per2* KO Zebrafish

**FIGURE 9:**
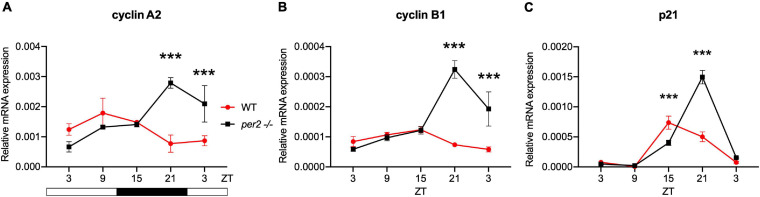
*Per2* knockout alters the rhythmic mRNA expression of cell cycle regulator genes in adult zebrafish fin tissues. qRT-PCR analysis of expression levels of some cell cycle CCGs **(A)** *cyclin A2*, **(B)** *cyclin B1*, and **(C)** *p21* in WT and *per2* KO fin tissue. Sets of 4 WT and *per2* KO fishes (each set containing two males and two females) were maintained under LD cycle (14 h light-10 h dark) conditions, a portion of caudal fin tissue was amputated and pooled for RNA extraction at 6-h intervals during a sampling window of 24 h. Mean mRNA relative expression (*n* = 2–3) ± SD is plotted on the *y*-axis; *zeitgeber* time (ZT) is plotted on the *x*-axis. *Zeitgeber* times are indicated for each sample. ZT0 corresponds to lights-on, ZT14 to lights-off. A change in rhythm was observed for *cyclin A2*, *cyclin B1*, and *p21*, as determined by a significant ANOVA Genotype × Time interaction effect for all genes (*p* < 0.001, see Table 1). Asterisks represent levels of significance in comparing the two genotypes at each time point individually, corrected by Sidak’s method (***p < 0.001).

Given the proposed role of *per2* as a tumor suppressor gene we next investigated the contribution of the *per2* gene to the circadian regulation of the cell cycle in zebrafish. We tested the gene expression of the clock-controlled cell cycle checkpoint regulators *cyclin A2, cyclin B1* and *p21* in WT control and *per2* KO fins sampled *in vivo*. *p21* is a potent cyclin-dependent kinase inhibitor (CKI) which functions as a regulator of cell cycle progression from G1 to S phase, while *cyclin B1* and *cyclin A2* serve as regulators of the entry into M and S phase, respectively. In all cases, in the mutant samples we observed significant changes in the rhythmic profile of gene expression with a phase delay compared to WT fin controls (Figure 9).

**FIGURE 10:**
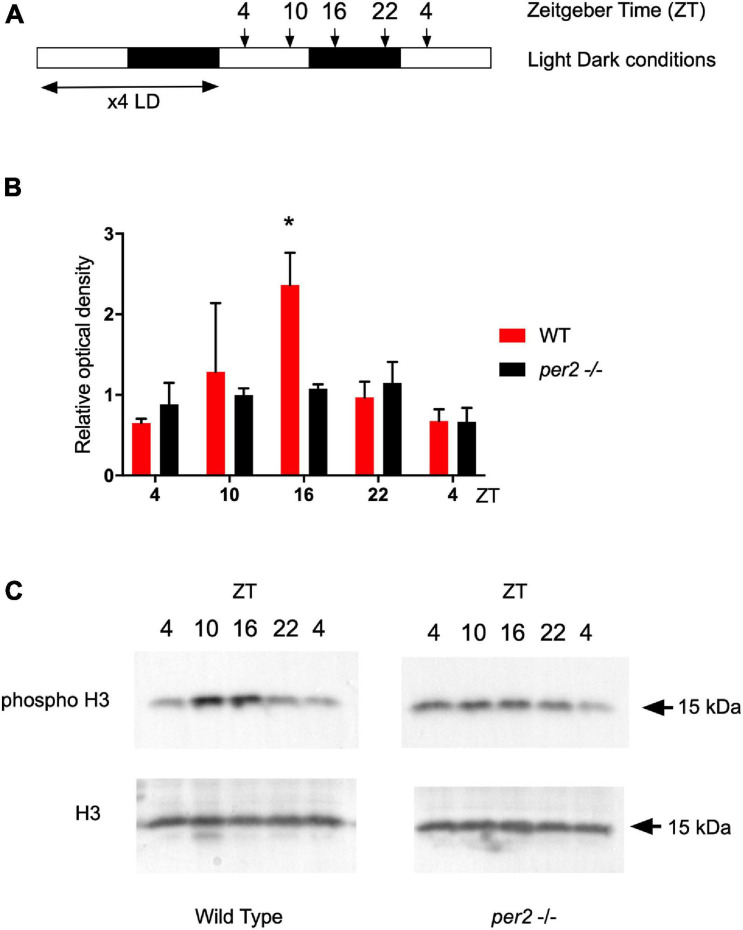
Western blot analysis and quantification of the phospho-H3 protein in fins of the WT and *per2* KO zebrafish line. **(A)** Schematic representation of the experimental design. **(B)** Quantification of Western blot analysis of Phospho-Histone H3 (p-H3) ser10 expression level normalized to using Histone H3 (H3) as loading control. Relative optical density values for pH3-specific bands were calculated from measurements of ECL images and plotted on the *y*-axis. **(C)** Representative images of western blots obtained with pH3 and H3 specific antibodies used for the quantification in panel **(B)**. Asterisks represent levels of significance in comparing the two genotypes at each timepoint individually, corrected by Sidak’s method (*p < 0.05).

In our previous studies, we have shown that M phase progression in zebrafish adult fin tissues, is gated to occur preferentially during the dark phase as a result of circadian clock regulation (Idda et al., 2012). Therefore, we tested whether dynamic changes in the levels of M phase were affected in the *per2* KO zebrafish adult fin tissues. We used a western blot assay to quantify levels of the phospho-H3 protein, a marker of chromatin compaction associated with mitosis, in the whole fin protein extracts of the WT and *per2* KO zebrafish lines. In agreement with our previous results (Idda et al., 2012), WT fin tissues exhibited a peak of phospho-H3 protein levels around ZT16 (Figure 10). Instead, in the *per2* KO fin tissues this peak was significantly reduced, consistent with abnormal circadian clock regulation of cell cycle progression in the *per2* KO proliferative fin tissues.

## Discussion

The data presented in this study point to a complex role for the *per2* gene in the circadian timing system in fish. In particular, we have demonstrated that the *per2* gene loss-of-function mutation affects rhythmic behavior of zebrafish larvae, the phase of rhythmic core clock gene expression, as well as the expression of certain CCGs in a tissue-specific manner. Finally, we reveal that loss of *per2* gene function is associated with abnormalities in the circadian regulation of the cell cycle *in vivo*.

### *Per2* Function Influences Clock-Controlled Behavior

The light inducible expression of the *per2* gene in the mammalian SCN as well as in the pineal gland, brain and peripheral tissues in fish point to an important role for the *per2* gene in the entrainment of the circadian clock by light. This prediction is supported here by the difference between WT and *per2* KO larvae in the amplitude and robustness of their locomotor activity rhythms in response to a 3-h light pulse. Nevertheless, the fact that the clock of *per2* KO fish is still somewhat entrained by light indicates the existence of mechanisms which compensate for the loss of *per2* gene function. It is tempting to speculate that the clock gene *cry1a* may be an element of such a mechanism. *Cry1a* is another robustly light-induced clock gene which is able to interact with other core clock proteins, such as CLOCK and BMAL (Tamai et al., 2007). In addition, we have previously shown that 1-h exposure to light is sufficient to induce the expression of several core clock genes or accessory clock genes (Ben-Moshe et al., 2014) suggesting the existence of additional compensatory factors.

In addition to light-entrainment, *per2* loss-of-function also strongly affects the phase of the locomotor activity rhythms, suggesting a role for PER2 in the clock mechanism itself. Interestingly, the effect of *per2* KO on the circadian phase is context-dependent – under constant dim-light *per2* KO led to a phase delay of 2.7 h while under constant light *per2* KO led to a phase advance of 2.3 h. Since zebrafish *per2* is a light-responsive gene, the light-dependence of *per2* KO effects may be predicted. It is tempting to speculate that the different phase shifts observed under different light intensities in the mutant might reflect accompanying differences in light-induced signal transduction in the larvae which subsequently differentially target PER2 function. Furthermore, the phase of the locomotor activity rhythms in the mutants shows a higher variability relative to WT controls pointing to a disruption of the entire clock system by the *per2* mutation. Thus, using rhythmic locomotor activity as a clock output we have demonstrated that *per2* is involved in both the core clock mechanism and its entrainment by light.

### *Per2* Regulation of Clock Gene Expression

Period proteins together with the cryptochromes are classically regarded as negative elements in the transcription translation feedback loop mechanism at the core of the circadian clock (Partch et al., 2014). However, the lack of a major effect on cycling clock gene expression in *per2* KO larvae would tend to argue against such a global role for PER2 in zebrafish. We did observe significant tissue-specific differences in the dynamic expression pattern of core clock genes in our *per2* KO fish. For example, rhythmic expression of *clock1* mRNA was affected in the liver, heart, muscle and gut of the *per2* KO, but not in other tissues. PER2 shares amino acid sequence motifs with both coactivators and corepressors of hormone receptors. For example, the mouse PER2 protein is characterized by two LXXLL motifs in both of its predicted protein–protein interaction domains (Albrecht et al., 2007). This motif is present in different coactivators which interact with nuclear receptors such as the steroid hormone receptor coactivator-1 (SRC-1) (Oñate et al., 1995). Moreover, it has been demonstrated that the PER2 protein upregulates *bmal1b* gene expression by directly binding to the Rorα nuclear receptor in zebrafish (Wang et al., 2015). Interestingly, a ROR/REV-ERB response element (RORE) has also been identified in the zebrafish *clock1* promoter, an observation that would potentially account for the dysregulation of *clock1* gene expression in certain tissues of *per2* KO adults. Thus, light-induced *per2* expression might serve to adjust the phase and amplitude of rhythmic expression of the *clock* gene. An unresolved issue remains how the observed changes in the profile and timing of *clock1* and *cry1a* mRNA expression in certain tissues does not lead to corresponding alterations in the rhythmic expression of other core clock component genes, such as *per1b* as well as other CCGs. It is tempting to speculate that the effects of PER2 are manifest in a gene-specific fashion and may reflect the different constellations of clock-regulated enhancer elements present in the promoters of various CCGs and core clock genes.

### *Per2* Tissue-Specific Function

The contribution of *per2* to shaping the profile of tissue specific rhythmic core clock gene expression raises the question of the extent to which the *per2* gene may also play a role in regulating tissue-specific physiology. In order to address this question, we also analyzed CCG expression in the liver, heart and muscle of the *per2* KO line. In particular, we focused attention on the expression of genes that are involved in the regulation of molecular mechanisms which underlie important physiological processes ranging from metabolism, development and maintenance of homeostasis to basic cellular processes, including cell growth and division (proliferation), cell movement (migration), controlled cell death (apoptosis) and cell differentiation. In the *per2* KO heart we observed a significant impact on the circadian expression of the *timp3, mef2a, cox6a2*, and *smad3a* genes (Wang et al., 2005, 2007; Sloin et al., 2018). Our results therefore implicate *per2* in the circadian clock-mediated regulation of various cardiac functions. This is of potential medical importance because there is a well-documented time of day-dependent increase in the sensitivity to myocardial infarction (Peckova et al., 1998) and disruption of circadian rhythms is a major contributor to heart pathophysiology (Crnko et al., 2019).

We also investigated the involvement of the *per2* gene in the circadian clock regulation of liver-specific CCGs, such as *impdh2, cyp1a*, *ppargc1b*, and *hnf1a* as well as genes encoding rate-limiting enzymes involved in the biosynthetic pathways for non-essential amino acids, namely *got1*, *got2a*, *asns*, *glud1b, glu1a*, and *gpt2l*. The results reveal an impact of the *per2* mutation on the rhythmic expression of many of these key metabolic regulatory-specific genes. Interestingly, the *per2* gene has already been associated with the regulation of liver-specific metabolic pathways in mammals. In particular, REV-ERBα and PPARα interact with the PER2 protein in the liver to regulate the transcription of their target genes (Schmutz et al., 2010). Moreover, using *per2* KO mice, it has been shown that PER2 directly represses the nuclear receptor PPARγ, critical for adipogenesis, and hepatic insulin sensitivity (Grimaldi et al., 2010). Therefore, these previous findings together with our own CCG expression analysis support the notion that the *per2* gene plays an important role in liver physiology. We also reveal disruption of rhythmic expression of the CCGs *myf6* and *hsf2* in the *per2* KO skeletal muscle. Thus, PER2 may play an important role in the temporal coordination of the mechanisms which direct the repair of muscle damage generated during daytime-elevated locomotor activity.

### *Per2* Gene Function Influences Circadian Clock Regulation of the Cell Cycle

The involvement of *per2* gene function in the circadian regulation of the cell cycle has been widely demonstrated in mammals (Fu et al., 2002; Gu et al., 2012; Yang et al., 2012; Tan et al., 2015). Consistently, we observed a robust effect of the loss of *per2* gene function on the phase of rhythmic expression pattern of important cell cycle regulators, *p21, cyclin A2*, and *cyclin B1* in adult fin tissue. It is important to note that in the same tissue, rhythmic expression of clock genes is apparently normal indicating that the *per2*-regulated expression of clock-controlled cell cycle regulators in peripheral tissues may be regulated by a distinct mechanism from the transcriptional control circuits within the core circadian clock mechanism itself. Namely, the cell cycle control represents a specific, clock output function for *per2*. In the case of the *p21* gene in zebrafish, transcriptional regulation by the core clock mechanism via E-box enhancers has been shown to direct rhythmic expression (Laranjeiro et al., 2013). Furthermore, at a mechanistic level, it has already been demonstrated that the PER2 protein modulates p53 stability and transcriptional activity in normal human cells, thus affecting the gene expression of cell cycle regulators, including *p21*, in response to DNA damage in mammals (Gotoh et al., 2014). Therefore, the phase-setting effects on *p21* rhythmic expression may rely on the loss of protein-protein interaction between PER2 and p53 in the *per2* KO zebrafish line. Previously, it has been shown that M phase progression in zebrafish adult fin tissue, shows a light-entrained, circadian clock regulation (Idda et al., 2012). Our quantification of the levels of mitosis throughout the LD cycle, revealed a dampened M-phase rhythm in the *per2* KO fin tissue. The dysregulation of rhythmic M phase progression, together with the abnormal gene expression profile of *p21*, *cyclin A2*, and *cyclin B1*, indicates that *per2* may play a role in timing of the G1/S or G2/M cell cycle checkpoints. Given the impact of *per2* gene function on the regulation of the expression levels of these important cell cycle regulators, it could be anticipated that PER2 may play a key role during the early stages of zebrafish embryonic development. However, the normal early embryonic development observed in *per2* KO mutants would tend to argue against this. Instead, this observation would tend to support a hypothesis that the contribution of PER2 to cell cycle regulation is cell type-or developmental stage-specific.

In conclusion, our results suggest that the *per2* gene plays a crucial role in the circadian regulation of multiple tissue-specific cellular and physiological processes in zebrafish.

## Data Availability Statement

The raw data supporting the conclusions of this article will be made available by the authors, without undue reservation.

## Ethics Statement

The animal study was reviewed and approved by the National Council for Animal Experimentation, Ministry of Health, Israel Local Government of Baden-Württemberg, Karlsruhe, Germany.

## Author Contributions

DV, NF, and YG designed the experiments. GR, ZB-ML, NG, and YW performed and interpreted the experiments. GR, ZB-ML, DV, YW, YG, and NF prepared the manuscript. All authors contributed to the article and approved the submitted version.

## Conflict of Interest

The authors declare that the research was conducted in the absence of any commercial or financial relationships that could be construed as a potential conflict of interest.

## Publisher’s Note

All claims expressed in this article are solely those of the authors and do not necessarily represent those of their affiliated organizations, or those of the publisher, the editors and the reviewers. Any product that may be evaluated in this article, or claim that may be made by its manufacturer, is not guaranteed or endorsed by the publisher.
