# Supplementary material for: Period 2: A Regulator of Multiple Tissue-Specific Circadian Functions
Source: Front Mol Neurosci. 2021 Sep 3;14:718387. doi: 10.3389/fnmol.2021.718387 (PMC8446368; doi:10.3389/fnmol.2021.718387)
Supplement: Supplementary file 1 [file Table_1.DOCX]

**Supplementary Table 1**

qRT-PCR primer sequences.

| **zf β-actin** | F: GCCTGACGGACAGGTCAT | R: ACCGCAAGATTCCATACCC |
| --- | --- | --- |
| **zf per1b** | F: CCGTCAGTTTCGCTTTTCTC | R: ATGTGCAGGCTGTAGATCCC |
| **zf clock1** | F: CTGGAGGATCAGCTGGGTAG | R: CACACACAGGCACAGACACA |
| **zf cry1a** | F: CAAACACTGCAGCAAAAACC | R: TCCGCTGTGTGTACATCCTC |
| **zf timp3** | F: GCTGGGAGCATCTCTCACTG | R: CGTAGTGGCGTGACTGGTAG |
| **zf mef2a** | F: GGCTCTCCAGGGCTCTCTAT | R: CATTCTGGCTGGTGTTGATG |
| **zf cox6a2** | F: GGCAAACGTTTACCTGAAGATG | R: TCAGTGATGAGGGCCTTCA |
| **zf smad3a** | F: ACCAAACCCTGTGTCTCCTG | R: GCTGTGAGGCATGGAAAGTT |
| **zf impdh2** | F: TGCCGTCTGCTGTTTGTATC | R: CCGGAGTGAAATGGTCTGT |
| **zf hnf1a** | F: ATTGCCCCAAGCTCCTTTAT | R: TACTGCTGTCTGCGATCACC |
| **zf cyp1a** | F: AAACCAGTGGCAAGTCAACC | R: AAAACCAACACCTTCTCGCC |
| **zf ppargc1b** | F: TGTCCTGTTCACCTCCTTCC | R: TCCATGACACGTCTCTGAGC |
| **zf glu1a** | F: GAAATGCGGGAAGATGGTGG | R: CAGTGAGTCGACGAGCATTG |
| **zf asns** | F: AGGAGCACATCGAGTCTGAG | R: CTTGGCCAGGGTAATGCTTC |
| **zf gpt2l** | F: GGGTCCCGAGTACTCCAAAA | R: GCTTTCACATCCGCATCCAT |
| **zf glud1b** | F: CAACACCCGATGCTGACAAA | R: AGCAGGTGGTAGTTGGAGTC |
| **zf got1** | F: TCACACTAAACACCCCGGAA | R: GAGTTCCCAGAGCCTTCAGT |
| **zf got2a** | F: GGAGGCTTCACAGTGGTTTG | R: AGCCATGCCTTTTACCTCCT |
| **zf hsf2** | F: CCTTCTGGGCAAAGTTGAGCTG | R: GCTGCTTGTCTGTGTTTTCTGAATC |
| **zf myf6** | F: CAACGAAGCTTTTGACGCG | R: AACACGGCTCCTTCTCTATGACC |
